# Supplementary material for: Night Warming Has Mixed Effects on the Development of the Fall Armyworm, Spodoptera frugiperda (Lepidoptera, Noctuidae), in Southern China
Source: Insects. 2024 Mar 7;15(3):180. doi: 10.3390/insects15030180 (PMC10971192; doi:10.3390/insects15030180)
Supplement: Supplementary file 1 [file insects-15-00180-s001.zip › insects-2816426-supplementary.pdf]

Supplemental file

Details of *Spodoptera frugiperda* artificial diet

Eight months after invading China, Chinese Researchers made artificial diet for rearing *Spodoptera frugiperda* [1]. *S. frugiperda,* have been reared in lab for more than 4 years on this diet. We followed the steps described in Li et al (2019) to produce the artificial diet used in our work. Here, we list the components of the diet (Part 1) and its formulation (Part 2) in detail.

Part 1

Detailed prescriptions of artificial diet were listed in Table 1. Table 2 and Table 3 including the detailed ingredients of Multi-vitamins B and Wesson's salt listed in Table 1.

Table 1 Prescriptions of artificial diet for fall armyworm *Spodoptera frugiperda*

Components	Quantity(g)	Components	Quantity(g)
**Part A**		Ascorbic acid	1.50
Maize powder	150.00	Cholesterol	0.48
Soybean powder	87.00	Inositol	0.17
Yeast powder	30.00	Sorbic acid	1.00
Casein	15.00	Choline chloride	0.70
Sucrose	10.00	Methyl parahydrobenzoate	1.40
Distilled water	300.00	Multi-vitamins B	0.50
**Part B**		Wesson's salt	0.25
Agar powder	15.00	Distilled water	35.00
Distilled water	350.00	**Part D**	
**Part C**		Colza oil(ml)	2.00

Table 2 The detailed composition of multi-vitamin B in the artificial diet

Ingredients	Quantity(g)
Nicotinamide	0.1525
Thiamine hydrochloride	0.0382
Riboflavin	0.0764
Pyridoxine hydrochloride	0.0382
Cyanocobalamin	0.0010
Folic acid	0.0382
D-Pantothenic acid calcium salt	0.1528
D-(+)-Biotin	0.0305

Table 3 The detailed composition of Wesson's salt in the artificial diet

Ingredients	Quantity(g)
NaCl	10.500
KCl	12.000
KH_2PO_4	31.000
$Ca_3(PO_4)_2$	14.900
$CaCO_3$	21.000

$MgSO_4$	9.000
$FePO_4 \cdot 4H_2O$	1.470
$MnSO_4$	0.020
$K_2Al_2(SO_4)_4 \cdot 24H_2O$	0.009
$CuSO_4 \cdot 5H_2O$	0.039
NaF	0.057
KI	0.005

Part 2

The producing method for the artificial diet for fall armyworm, *Spodoptera frugiperda*, follows a precise sequence of steps:

(1) Accurately weigh the components in Tables 1, 2, and 3 for later use.

(2) Place thoroughly stirred components A and B separately in a high-pressure steam sterilization pot and sterilize at 121 ℃ for 20 minutes. After sterilization, pour component B into component A and stir vigorously, then cool to about 60 ℃ for later use.

(3) Put the weighed component C into a beaker, add 50ml warm distilled water, stir and dissolve while stirring. After it is completely dissolved, pour it into the diet in step (2), then add component D, and stir until it is completely uniform.

(4) Pour the uniformly mixed diet into a fresh-keeping box. Allow the diet to completely cool and solidify. Once solidified, seal the container securely and store it in refrigerator at 4 ℃.

References

1. Li, C. Y.; Zhang, Y. P.; Huang, S. H.; Liu, W. L.; Zhang, Y. P. Study on indoor captive rearing technology of fall armyworm. *China Journal of Environmental Entomology* **2019**, 41, 986-991. DOI: 10.3969/j.issn.1674－0858.2
